# Three-dimensional-printed porous implant combined with autograft reconstruction for giant cell tumor in proximal tibia

**DOI:** 10.1186/s13018-021-02446-x

**Published:** 2021-04-29

**Authors:** Yuqi Zhang, Minxun Lu, Li Min, Jie Wang, Yitian Wang, Yi Luo, Yong Zhou, Hong Duan, Chongqi Tu

**Affiliations:** 1 Department of Orthopedics, Orthopedic Research Institute, West China Hospital, Sichuan University, No. 37 Guoxuexiang, Chengdu, 610041 Sichuan People’s Republic of China; 2 Bone and Joint 3D-Printing & Biomechanical Laboratory, Department of Orthopedics, West China Hospital, Sichuan University, No. 37 Guoxuexiang, Chengdu, 610041 Sichuan People’s Republic of China

**Keywords:** 3D printed, Prosthesis, Giant cell tumor, Proximal tibia

## Abstract

**Background:**

This study is to describe the design and surgical techniques of three- dimensional-printed porous implants for proximal giant cell tumors of bone and evaluate the short-term clinical outcomes.

**Methods:**

From December 2016 to April 2020, 8 patients with giant cell tumor of bone in the proximal tibia underwent intralesional curettage of the tumor and reconstruction with bone grafting and three-dimensional-printed porous implant. Detailed anatomy data were measured, including the size of lesion and thickness of the subchondral bone. Prostheses were custom-made for each patient by our team. All patients were evaluated regularly and short-term clinical outcomes were recorded.

**Results:**

The mean follow-up period was 26 months. According to the different defect sizes, the mean size of the plate and mean length of strut were 35 × 35 mm and 20 mm, respectively. The mean affected subchondral bone percentage was 31.5%. The average preoperative and postoperative thickness of the subchondral bone was 2.1 mm and 11.1 mm, respectively. There was no wound infection, skin necrosis, peroneal nerve injury, or other surgical related complications. No degeneration of the knee joint was found. Osseointegration was observed in all patients. The MSTS improved from an average of 12 preoperatively to 28 postoperatively.

**Conclusion:**

The application of three-dimensional-printed printed porous prosthesis combined autograft could supply enough mechanical support and enhance bone ingrowth. The design and operation management lead to satisfactory subchondral bone reconstruction.

## Background

Giant cell tumor of bone (GCTB) is a benign but aggressive tumor mostly affecting the bones around the knee [1]. The epiphyseal region of the proximal tibia is the second most common location after the distal femur [2]. According to Campanacci Grade of GCTBs, extended intralesional curettage with osseous voids filling is the primary surgical option for grades I and II GCTBs in proximal tibia [1, 3]. Despite the mechanical support, osseous voids filling after curettage frequently resulted in mechanical failures, including deformity, fractures, and even collapse of the articular surface. Recent evidence suggests that the damage or destruction of the subchondral bone (SCB) accounts for these mechanical failures to a certain degree [4]. Thus, when the SCB was severely damaged, the protection and reconstruction of SCB were considered the most crucial principles for treating GCTs with Campanacci grades I and II around the knee. Moreover, the reconstruction methods for voids filling are closely related to the protection and promotion of the SCB.

Currently, bone cement and bone graft-assisted plate, Steinmann pins, or screws have all been utilized to fill the voids [5–10]. Cement packing was initially the most popular method because of its convenience. Besides, the thermal effect during cement hardening could extend the tumor kill zone and improve structural stability to some extent. However, the possible thermal necrosis damage to the SCB and the lack of bone inducibility and conductibility constitute a significant disadvantage [11, 12]. Bone graft was widely applied owing to the excellent biocompatibility. Nevertheless, the difficulty of detecting local recurrence and the weak mechanical strength limit its use [13]. In recent years, the combination of bone cement and autograft was one of the most respected methods, which is famous as “sandwich technique” [7]. However, the complications related to the non-biological bone-cement interface and the cytotoxic of cement have still not been solved.

Porous scaffold has been proved to promote bone and prosthesis interface integration [14, 15]. With the advantages of additive manufacturing, three-dimensional (3D) printed customized prosthesis with porous structures could be manufactured. Compared with the autograft, 3D-printed customized prosthesis combined with autograft could provide better biocompatibility and sufficient mechanical strength without cytotoxic or rejection reactions.

Based on our previous case report study, we designed optimized 3D-printed customized prostheses and applied them to treat patients with GCTB in the proximal tibia [12]. In this study, we described the experience of using 3D-printed customized prostheses for the defect reconstruction after extended intralesional curettage in the proximal tibia and evaluated the short-term clinical outcomes and complications.

## Method

### Patients

From December 2016 to April 2020, 8 patients (2 males and 6 females) with GCTB in the proximal tibia underwent intralesional curettage of the tumor and reconstruction with bone grafting and 3D-printed porous implant. Six patients were diagnosed with the primary tumor. One had previous resection surgery of the proximal fibula and suffered a recurrence that invaded the proximal tibia. The last one underwent curettage in previous surgery and suffered a local recurrence. Preoperative assessments included the examination of 100% magnified X-ray of the bilateral knee containing anteroposterior (AP) and lateral view, full-length radiograph of the lower limbs in a standing position, 3D-computerized tomography (CT) and magnetic resonance imaging (MRI) of the affected knee, thin-layer CT scan of the chest, and total body bone scan. The pathologic diagnosis was confirmed for each patient by needle biopsy or ex-surgery. 3D CT and MRI data were imported into the Mimics V22.0 software (Materialise Corp., Belgium).

**Fig. 1:**
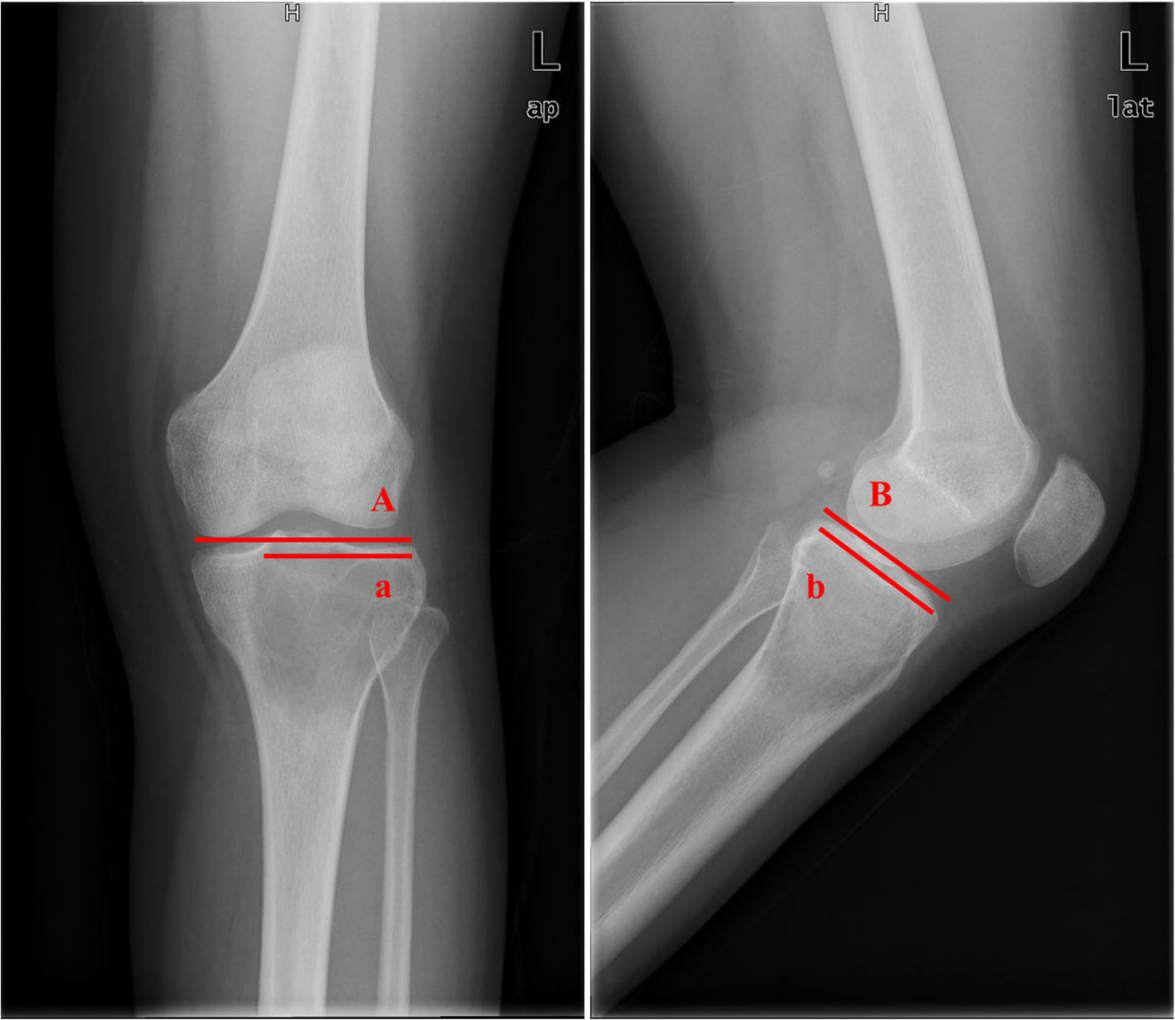
The calculation method of the affected SCB area proportion in the radiograph. A: length of total SCB on anteroposterior view; B: length of total SCB on lateral view; a: length of affected SCB on anteroposterior view; b: length of affected SCB on lateral view

The thinnest thickness of the subchondral bone was measured on the Mimics and recorded. The calculation method of the affected SCB area proportion was improved based on the method described by Chen [16]. The SCB was defined as invaded when there was less than a 3-mm distance to the tumor, and the invaded SCB was classified according to the thickness of residual SCB. Then, the lengths of the affected and total SCB were measured on the anteroposterior and lateral view of the X-ray. The area of the affected SCB of the proximal tibia was expressed as a percentage and was calculated as [a × b/(A × B)] × 100% (Fig. 1).

The surrounding bone quality and the safe border were evaluated according to MRI data on the Mimics. The lung metastasis and bone metastasis were accessed based on the lung CT scanning and total body bone scan, respectively. The pain at rest was precisely evaluated according to the visual analog scale (VAS). The knee joint function was recorded by the range of motion (ROM) and Musculoskeletal Tumor Rating Scale (MSTS) [17]. The osteoarthritis (OA) of the knee was graded through preoperative radiographs and the last follow-up radiographs according to the classification of Kellgren and Lawrence [18].

This study was approved by the Ethical Committee of our institution. Written informed consent was obtained from all patients when they agreed to use 3D-printed prosthesis.

### Prosthesis design

**Fig. 2:**
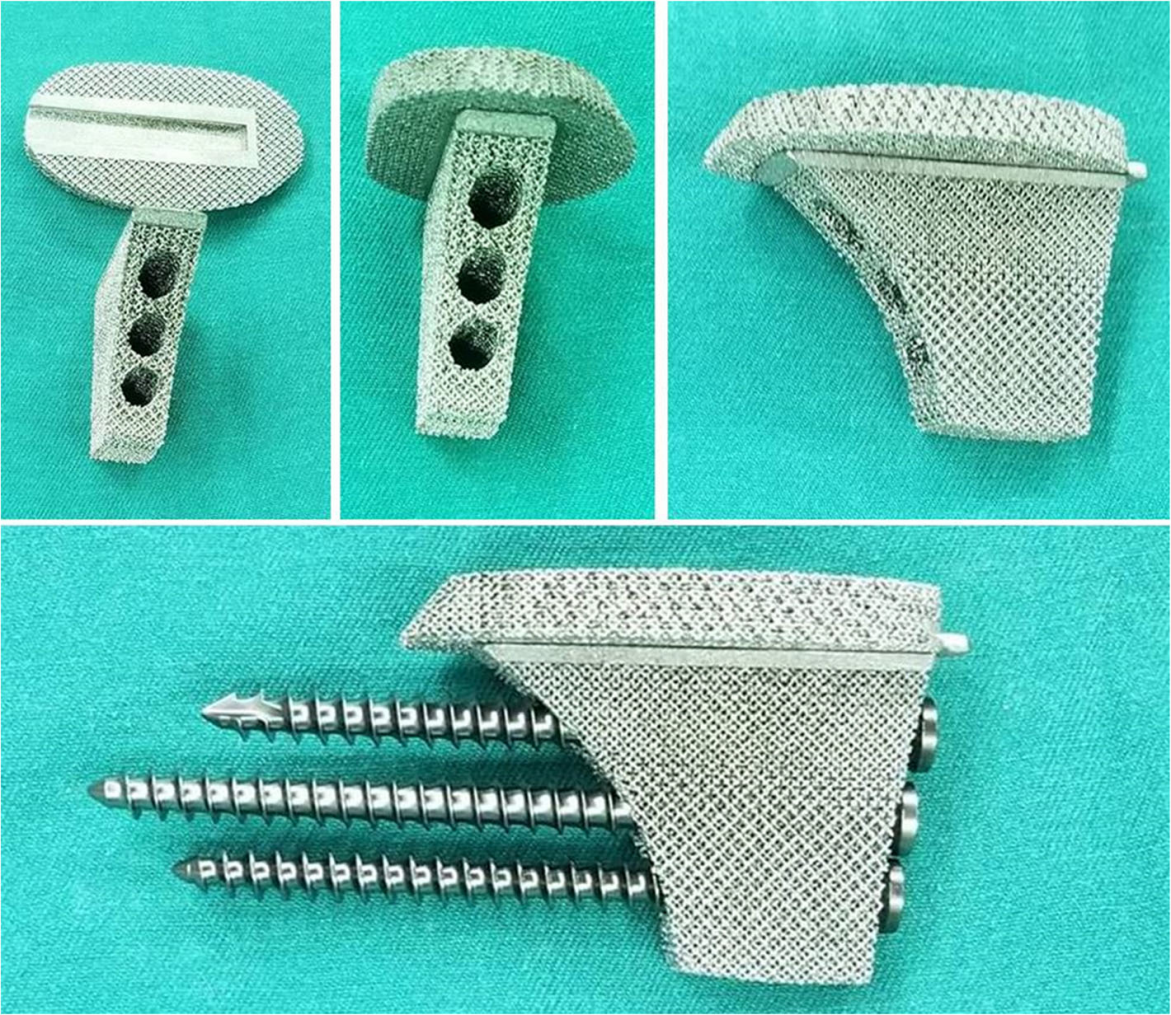
Prosthesis fabricated by electron beam melting technique

All prostheses were custom-made for each patient by our team with Solidworks 2016 (Dassault Systemes, France). All prostheses were made of titanium alloy and fabricated by Chunli Co., Ltd. (Tongzhou, Beijing, People’s Republic of China) with electron beam melting technique (ARCAM Q10plus, Mölndal, Sweden). The procedure of design and produce had been described thoroughly in our previous study [12]. The prostheses consisted of a porous truncated ellipsoid cone-shaped plate and a porous square frustum-shaped strut, which could be assembled by a specialized solid slideway. Three screw holes were added to the strut. Porous structure with 500-μm-pore size and 70% porosity was applied on the plate, and porous structure with 400-μm-pore size and 55% porosity was applied on the strut (Fig. 2). The prosthesis size was defined by length, width, and thickness.

### Surgical technique

**Fig. 3:**
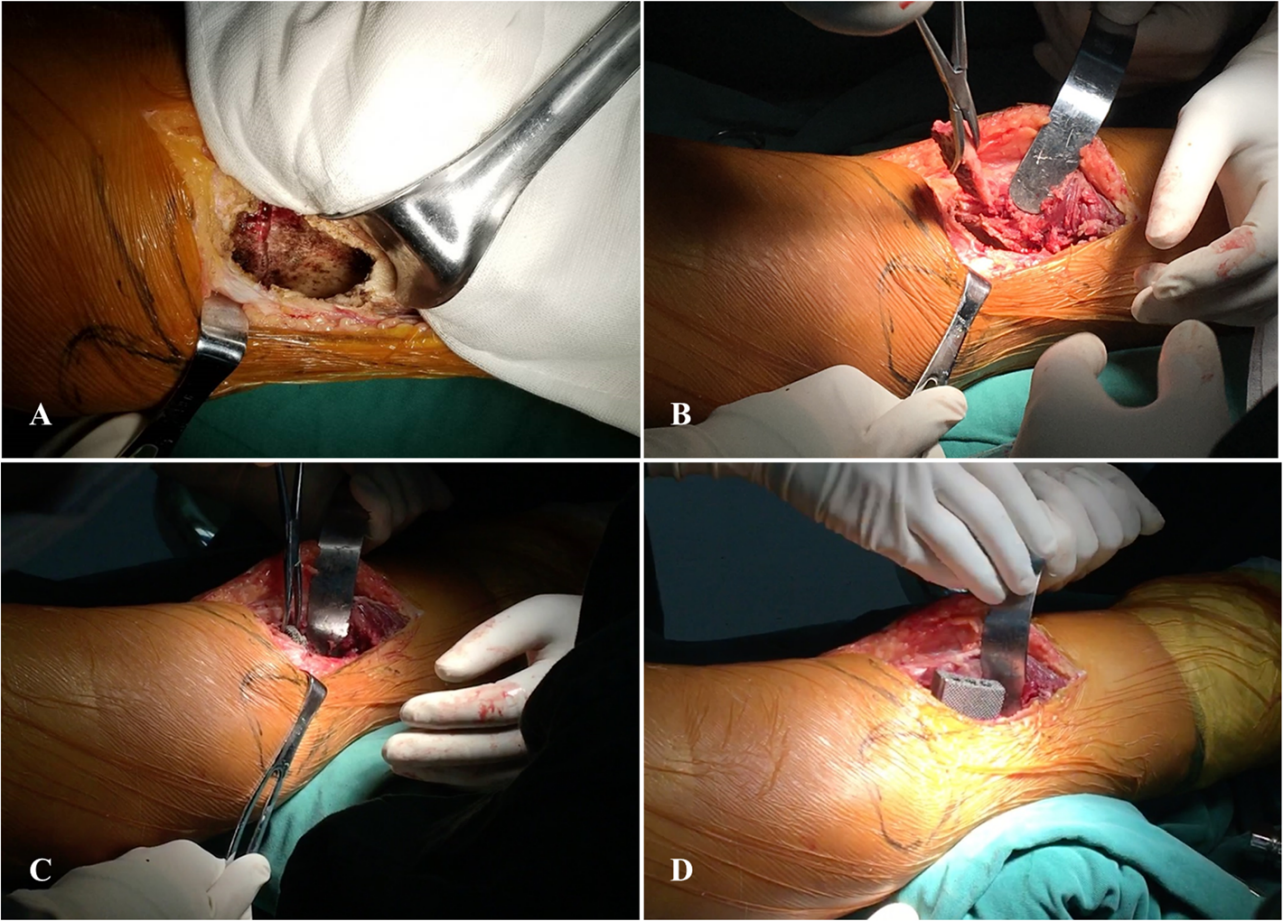
Intraoperative pictures. **a** Osseous void was rinsed clean after milling. **b** The decorticated autogenous iliac crest was placed under the subchondral bone. **c** The plate of prosthesis was placed vertically close to the iliac crest. **d** After aligning the slide way, the strut of prosthesis was implanted with hammering

All the surgeries were performed by the senior surgeon (Chongqi Tu) under general anesthesia. The patient was placed on the operation table in a supine position. The approach was selected according to the most severely affected condyle of the proximal tibia. A cortical window that was slightly smaller than the tumor margin was created with a mini drill. After the tumor was removed, a high-speed burr was used to achieve extensive intralesional curettage. Then, phenol, hydrogen peroxide solution, and pulsatile lavage with a large amount of saline solution were used to rinse the cavity. After that, an iliac crest autogenous bone graft with a decorticated surface was used to repair subchondral bone defects. Then, the bearing plate with the porous surface facing the iliac crest was inserted into the cavity, and the struct was implanted into the cavity through the sideway on the porous plate. Furthermore, three screws were fixed to the contralateral proximal tibia cortex to improve the initial stability. The residual cavity was filled with autogenous bone or artificial bone. Finally, all incisions were closed (Fig. 3).

### Postoperative management

For all patients, the affected limb was immobilized for 1 week. Passive and positive exercises without weight-bearing were recommended in the first 2 weeks. For patients whose SCB thickness less than 1.0 mm, partial weight-bearing standing was allowed 2 weeks post-operation, and walking with crutches should begin in the 3 weeks. Relatively, for patients whose SCB thickness more than 1.0 mm, partial weight-bearing standing and walking with two crutches were encouraged 2 weeks post-operation. Then, gradual full weight-bearing was allowed.

All patients were evaluated regularly (monthly in the first three months and then trimonthly) with the physical examination, radiographs, and Tomosynthesis-Shimadzu Metal Artefact Reduction Technology (T-SMART). The thickness of the reconstructed SCB was recorded as the shortest distance from the articular surface of the SCB perpendicular to the superior surface of the plate on the AP view. The incorporations of the autograft and implant, the implant, and the host bone were evaluated by T-SMART. The observation of the connection between the trabecular structures and the implant surface in T-SMART was considered good osseointegration. Degeneration and articular surface collapse were evaluated by radiographs. CT of the chest was used to determine metastasis every 3 months. At the last follow-up visit, the pain (VAS), the function (ROM and MSTS), the thickness of the SCB, and the OA stage were recorded.

## Results

At the end of the follow-up period, all these patients were alive without recurrence and metastasis. The average age was 43 years (range, 26–56 years) with a mean follow-up of 26 months (range, 16–38 months). Six patients occurred at left proximal tibia, and two occurred at the right proximal tibia. The affected site was 3 in the lateral, 1 in the media, and 4 affected both the lateral and media sides. According to Campanacci’s [14] staging system, there were 5 in grade II, 3 in grade III (Table 1).

**Table 1:** Basic data of the patients

Patients	Sex	Age	Stage	Site	Media/lateral	Follow-up (month)	Pathological fracture
1	M	44	II	L	Lateral	38	
2	F	26	II	L	Both	32	
3	F	51	II	L	Both	29	
4	F	33	II	L	Lateral	28	
5	F	33	III	R	Both	26	Y
6	M	32	III	R	Lateral	21	
7	F	44	III	L	Both	20	Y
8	F	56	II	L	Media	16	

The thickness of plate and strut was 10.0 mm and 12.0 mm, respectively. According to the different defect sizes, the mean size of the plate was 35 × 35 mm (mean length × mean width) and mean length of strut was 20 mm (range, 18–22 mm). Seven patients’ SCB thickness was less than 3 mm, and one patient’s SCB thickness was 4 mm. The mean affected SCB percentage was 31.5% (range, 10.8–59.5%). The average postoperative thickness of the SCB immediately after the surgery was 11.1 mm (range, 9.6–13.4 mm). At the last follow-up, the mean thickness of the SCB was 11.0 mm (range, 9.4–133.4 mm). Detailed data of SCB was listed in Table 2.

**Table 2:** Patients’ SCB data and function outcomes

Patients	Pre-SCB thickness	a	b	A	B	Percentage (%)	Pos-SCB thickness	Follow-up SCB thickness	MSTS (pre)	MSTS (pos)	ROM
1	1.1	19.9	90.8	28.7	58.2	10.8	10.9	10.9	9	26	0–115°
2	1.2	44.8	71.4	40.4	42.6	59.5	13.4	13.4	16	30	0–150°
3	3.3	55.1	76.3	32.5	46.2	50.8	9.6	9.4	17	30	0–150°
4	4.1	25.0	73.4	14.7	38.3	13.1	11.8	11.6	16	30	0–138°
5	3.2	56.2	87.0	45.1	51.9	56.1	12.2	11.8	4	27	0–139°
6	1.0	26.2	88.5	44.1	50.2	26.0	11.1	11.0	11	29	0–145°
7	0.4	41.2	73.3	21.3	51.7	23.2	12.1	11.5	5	22	0–140°
8	2.2	18.5	68.9	20.4	44.0	12.4	9.8	9.6	16	30	0–143°
Mean	2.1	35.9	78.7	30.9	47.9	31.5	11.1	11.0	12	28	0–140°

**Fig. 4:**
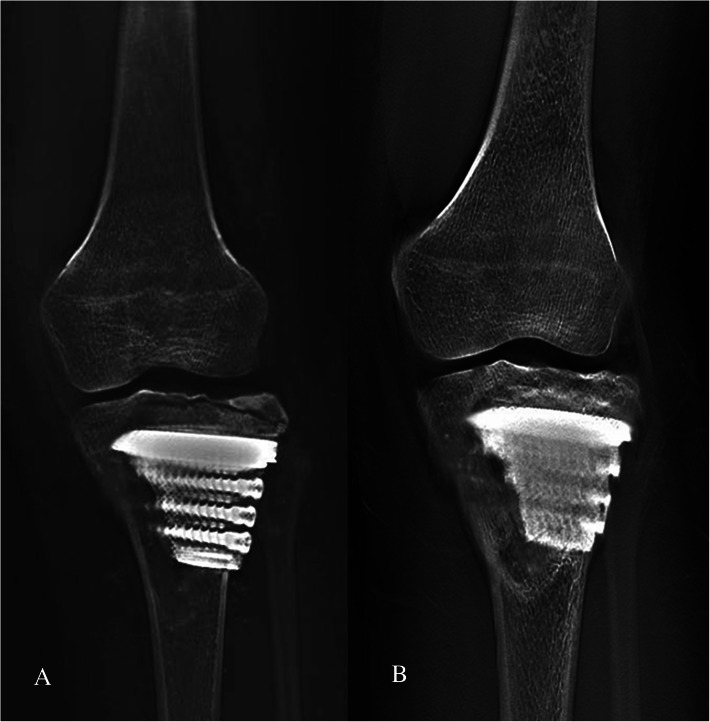
Postoperative T-SMART showed preliminary osseointegration. **a** One month after surgery. **b** Five months after surgery

**Fig. 5:**
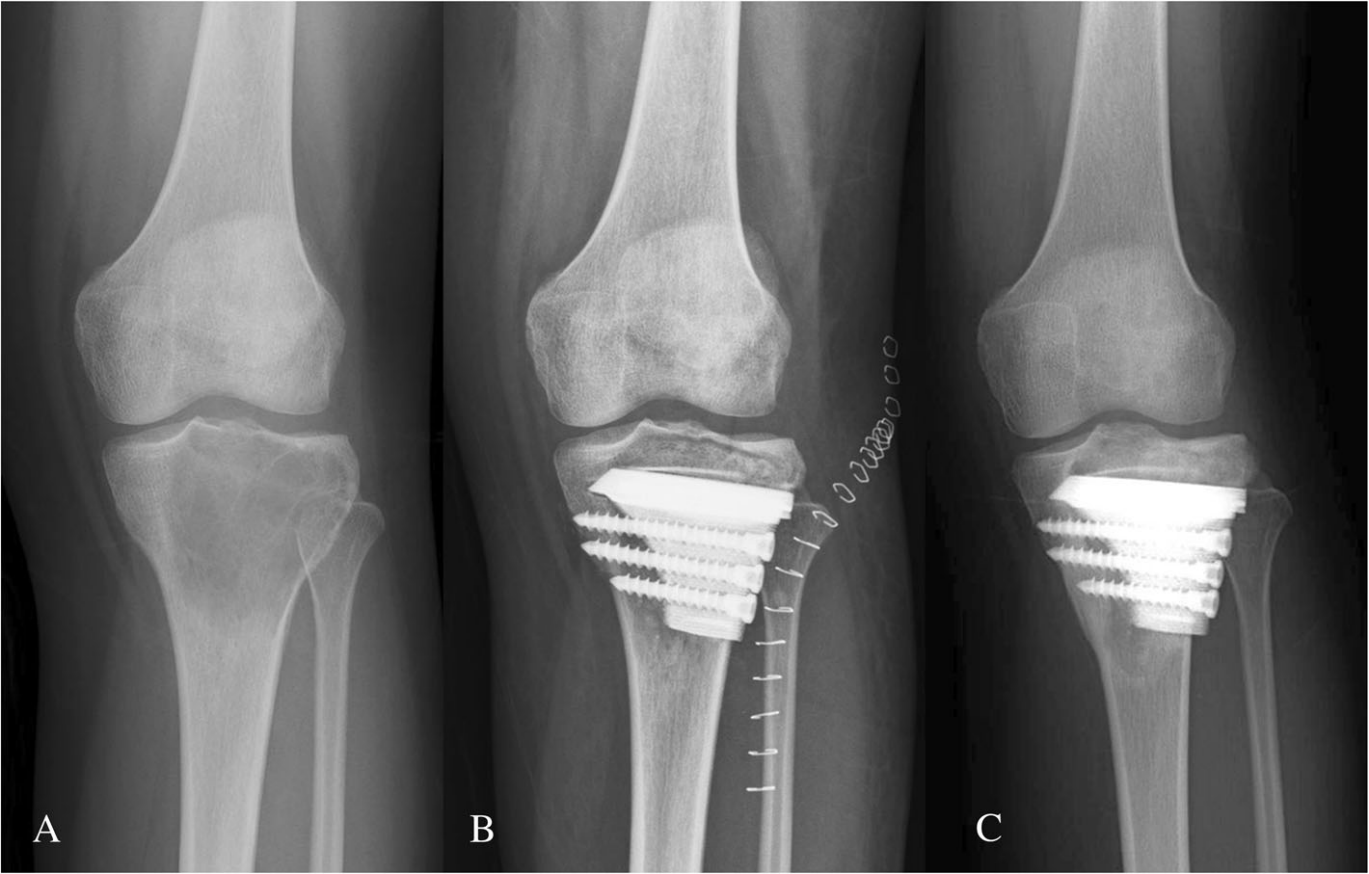
Comparison between preoperative and postoperative X-rays. **a** Before surgery. **b** One week after surgery. **c** One year after surgery

**Fig. 6:**
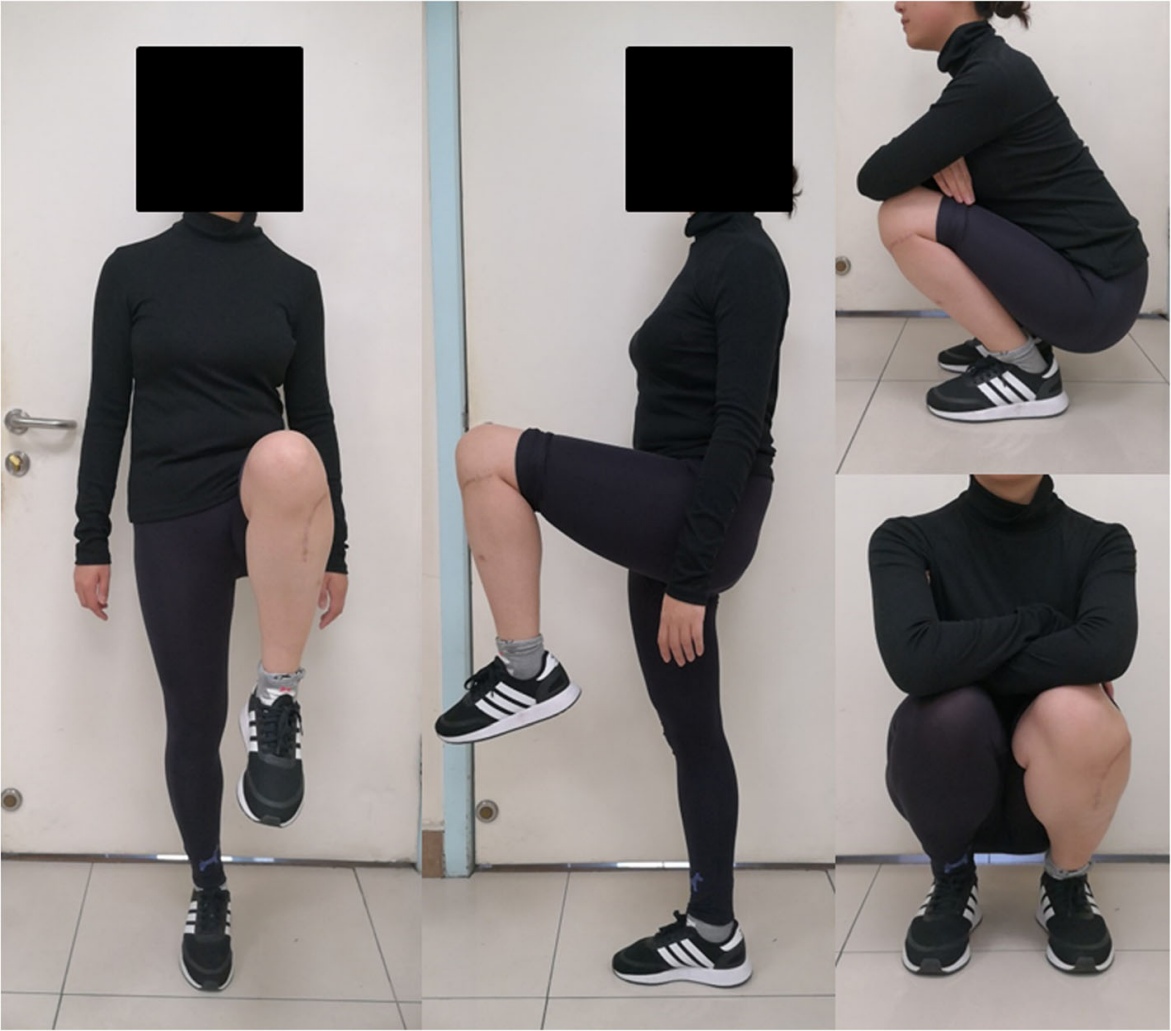
Six months after surgery, the knee joint function of one patient

There was no wound infection, skin necrosis, peroneal nerve injury, or other surgical-related complications (including the graft harvest site, thrombosis, and decubitus). No degeneration of the knee joint was found. Furthermore, there were no complications associated with prostheses, such as aseptic loosening or breakage. The autograft achieved bone union in all patients at a mean time of 3.6 months (range, 3–5 months). The absence of interfacial gap between prosthesis and bone was found in T-SMART average five months postoperatively, which was considered integration well (Figs. 4 and 5). The VAS decreased from a mean of 7 (range, 5–9) preoperative to 0 (range, 0–1) postoperatively. The MSTS improved from an average of 12 (range, 4–17) preoperatively to 28 (range, 28–30) postoperatively (Fig. 6).

## Discussion

The treatment of GCTBs in proximal tibia involving the subchondral bone is challenging, including reconstruction with polymethylmethacrylate bone cement or bone graft after extensive intralesional curettage and tumor knee arthroplasty after segmental resection. Because of the importance of knee joint in weight-bearing and many other activities, extensive intralesional curettage was preferred for GCTBs around the knee, which could preserve joint integrity and maximizes function [11, 19]. Niu et al. [1] followed those patients who had undergone segmental resection and extensive intralesional curettage. After a median duration follow-up of 13.7 months, the functional outcomes of curettage were significantly superior to those of resection.

After curettage, many surgeons pack the defect with cement or cement combined with bone graft. Currently, the combination of autogenous bone and bone cement was the most commonly accepted for the good biocompatibility of autogenous bone and good immediate stability of bone cement. However, some disadvantages of this method were inevitable. Firstly, the associated exothermic reaction during the hardening process of the bone cement could make the surrounding bone undergo necrosis [11], which decreased osteogenesis. Secondly, bone cement filling was a non-biological reconstruction and cannot be integrated with the autogenous bone to achieve osseointegration, which showed a clear translucent line around the cement visible on imaging. Zheng et al. [13] reported those patients with curettage and cement filling. After an average follow-up of 78.8 months, a radiolucent zone between the cement and cortical bone could be found in most patients at the six months after operation without width increase or disappearance. Some researches believed that the radiolucent zone might allow micromotion between bone cement and bone, which greatly increased the risk of SCB non-union and fractures [20, 21], thus caused articular surface collapse and knee osteoarthritis.

With the development in material science and manufacturing, 3D-printed porous titanium scaffold with excellent biocompatibility, bioactivity, and mechanical property brings a new approach to reconstruct bone defect. A previous study proved that porous scaffold with specific pore size and porosity could induce the bone ingrowth and achieve integration with bone tissue [22]. Thus, more and more 3D-printed porous titanium prostheses were applied to reconstruct bone defects. Compared to the polymethylmethacrylate bone cement, 3D-printed porous titanic prosthesis was made of bio-inert materials, which could avoid the associated exothermic reaction and osteonecrosis of bone cement. Also, the porosity and pore size could be customized to match the biomechanics of the proximal tibia. According to Torres-Sanchez’s research [23], mechanical properties of porous scaffolds with different ranges of porosities could mimic the mechanical properties of cortical and trabecular bone, respectively. Thus, our prosthesis’ porous strut and plate were designed with a pore size of 400 μm and 55% porosity and pore size of 500 μm and 70% porosity, respectively.

In our study, 3D-printed porous titanic prosthesis combined with the autograft was applied to reconstruct the bone defect and the SCB after extensive intralesional curettage. The average SCB thickness was improved to 11.1 mm from 2.1 mm. No degeneration of the knee joint was found, and satisfactory bone healing was achieved at the interface of prosthesis and bone in all patients after 3.6 months of implantation on average. At a mean of 5 months postoperatively, the absence of interfacial gap between endoprosthesis and bone was found in T-SMART, which was considered integration between bone and prosthesis. The postoperative function of knee joint was significantly improved compared with that preoperatively, and the pain was also significantly relieved. The favorable outcomes resulted from the excellent reconstruction of SCB and enough mechanical support of prosthesis. The study of Mahjoub et al. [24] showed that SCB was involved in osteoarthritis and had functional interactions between the bone and cartilage. Teng et al. [25] retrospectively reviewed 104 patients, and the result showed that if the subchondral bone layer was less than 3.3 mm, patients had a higher chance of mechanical failure postoperatively. A similar result was also reported by Chen et al. [16]. Therefore, a modified iliac crest bone was used to repair the SCB.

A suitable prosthesis design would be a crucial reason for better function with a low chance of complications. In order to reduce the window size and bone loss, the prosthesis was designed as two parts assembled by an inverted trapezoidal compression slide way for easier implantation and better stability. To distribute the weight-bearing stress evenly, the prosthesis plate was designed to be a slightly smaller ellipsoid than the defect to fit the shape of the tibial plateau. Besides, to balance the mechanical strength and the implantation convenience, based on our experiences, the most suitable thickness of the plate and the strut was 10 mm and 12 mm, respectively. The strut extended up to the plate and down to the inferior border of the bone defect, and the sloping design of the lower edge of the strut could provide axial compression during implantation to fit closer between the plate and the bone. Furthermore, three screw holes were designed for screws fixation to the contralateral bone cortex to enhance immediate stability.

Besides the design of prosthesis, the appropriate surgical technique was also crucial for good outcomes. Firstly, the size and location of window should be determined according to the tumor’s size and location. The width and length of window should be smaller than the tumor cavity so that it could enlarge to the most proper size when prosthesis was implanted. Secondly, the autogenous bone should be decorticated appropriately before implantation. The bone cortex has good mechanical strength, but it could block the bone ingrowth into the implant surface and heal with the host bone. Thus, the cortex of autogenous bone facing the SCB should be removed to promote the healing with the host bone, and the cortex of another side could decorticate partially to maintain mechanical strength to some degree. Besides, the decortication could be achieved by ichthyologizing with a file. Thirdly, the strut and plate of prosthesis must be implanted in a specific order. To reduce unnecessary bone loss, the weight-bearing plate of prosthesis could be implanted into the cavity vertically and rotate to the correct position. After that, the gaps between the plate and the autogenous bone should be filled with cancellous bone or artificial bone. When implanting the strut, the cortical window could be extended downwards according to the strut's length to obtain maximum initial stability.

Our study had some unavoidable limitations. Firstly, this study is retrospective and has a small sample size with a short-term follow-up, a control group, and long-term follow-up is required. Secondly, there is no biomechanical analysis included in our study; thus, finite element analysis should be done in the next step. Besides, the prosthesis design and fabrication take 1 to 2 weeks, which means patients have to wait. However, if denosumab can be used in China, patients can use denosumab while waiting for the prosthesis design and fabrication, which can obviously make up for this shortcoming.

## Conclusion

The application of 3D-printed porous prosthesis combined with autograft could supply enough mechanical support and enhance bone ingrowth. The minimum reconstructive thickness of SCB could be reduced effectively by this approach. The rational design and strict operation management lead to satisfactory SCB reconstruction with favorable osseointegration.

## Data Availability

The data and materials are available from the medical records department of the West China Hospital. The datasets used and analyzed during the current study are available from the corresponding author on reasonable request.

## Abbreviations

GCTB: Giant cell tumor of bone
SCB: Subchondral bone
3D: Three-dimensional
CT: Computed tomography
AP: Antero-posterior
MRI: Magnetic resonance imaging
VAS: Visual analog scale
ROM: Range of the motion
MSTS: Musculoskeletal Tumor Rating Scale
T-SMART: Tomosynthesis-Shimadzu metal artifact reduction technology
OA: Osteoarthritis
KL: Kellgren and Lawrence

## References

[1] Niu X, Zhang Q, Hao L, Ding Y, Li Y, Xu H, Liu W (2012). Giant cell tumor of the extremity: retrospective analysis of 621 Chinese patients from one institution. J Bone Joint Surg Am..

[2] Dahlin DC, Cupps RE, Johnson EW (1970). Giant-cell tumor: a study of 195 cases. Cancer..

[3] Raskin KA, Schwab JH, Mankin HJ, Springfield DS, Hornicek FJ (2013). Giant cell tumor of bone. J Am Acad Orthop Surg..

[4] Abdelrahman M, Bassiony AA, Shalaby H, Assal MK (2009). Cryosurgery and impaction subchondral bone graft for the treatment of giant cell tumor around the knee. HSS J..

[5] Szalay K, Antal I, Kiss J, Szendroi M (2006). Comparison of the degenerative changes in weight-bearing joints following cementing or grafting techniques in giant cell tumour patients: medium-term results. Int Orthop..

[6] Ayerza MA, Aponte-Tinao LA, Farfalli GL, Restrepo CA, Muscolo DL (2009). Joint preservation after extensive curettage of knee giant cell tumors. Clin Orthop Relat Res..

[7] Banerjee S, Sabui KK, Chatterjee R, Das AK, Mondal J, Pal DK (2012). Sandwich reconstruction technique for subchondral giant cell tumors around the knee. Curr Orthop Pract.

[8] Wu M, Yao S, Xie Y, Yan F, Deng Z, Lei J, et al. A novel subchondral bone-grafting procedure for the treatment of giant-cell tumor around the knee. Medicine. 2018;97(45):e13154. 10.1097/MD.0000000000013154 PMC6250490 30407342

[9] Bini SA, Gill K, Johnston JO (1995). Giant cell tumor of bone. Curettage and cement reconstruction. Clin Orthop Relat Res.

[10] Ruskin J, Caravaggi P, Beebe KS, Corgan S, Chen L, Yoon RS, Patterson FR, Hwang JS (2016). Steinmann pin augmentation versus locking plate constructs. J Orthop Traumatol..

[11] Montgomery C, Couch C, Emory CL, Nicholas R (2019). Giant Cell Tumor of Bone: Review of Current Literature, Evaluation, and Treatment Options. J Knee Surg..

[12] Lu M, Wang J, Tang F, Min L, Zhou Y, Zhang W, Tu C (2019). A three-dimensional printed porous implant combined with bone grafting following curettage of a subchondral giant cell tumour of the proximal tibia: a case report. BMC Surg..

[13] Zheng K, Yu XC, Hu YC, Wang Z, Wu SJ, Ye ZM, Giant Cell Tumor Group of China (GTOC) (2017). How to Fill the Cavity after Curettage of Giant Cell Tumors around the Knee? A Multicenter Analysis. Chin Med J (Engl)..

[14] Ding X, Liu X, Chen J, Chen S (2018). Research progress of porous tantalum in bone tissue engineering. Zhongguo Xiu Fu Chong Jian Wai Ke Za Zhi..

[15] Chen Y, Frith JE, Dehghan-Manshadi A, Attar H, Kent D, Soro NDM, Bermingham MJ, Dargusch MS (2017). Mechanical properties and biocompatibility of porous titanium scaffolds for bone tissue engineering. J Mech Behav Biomed Mater..

[16] Chen TH, Su YP, Chen WM (2005). Giant cell tumors of the knee: subchondral bone integrity affects the outcome. Int Orthop..

[17] Enneking WF, Dunham W, Gebhardt MC, Malawar M, Pritchard DJ (1993). A system for the functional evaluation of reconstructive procedures after surgical treatment of tumors of the musculoskeletal system. Clin Orthop Relat Res..

[18] Kellgren JH, Lawrence JS (1957). Radiological assessment of osteo-arthrosis. Ann Rheum Dis..

[19] Errani C, Ruggieri P, Asenzio MA, Toscano A, Colangeli S, Rimondi E (2010). Giant cell tumor of the extremity: A review of 349 cases from a single institution. Cancer Treat Rev..

[20] Benevenia J, Rivero SM, Moore J, Ippolito JA, Siegerman DA, Beebe KS, Patterson FR (2017). Supplemental bone grafting in giant cell tumor of the extremity reduces nononcologic complications. Clin Orthop Relat Res..

[21] Wada T, Kaya M, Nagoya S, Kawaguchi S, Isu K, Yamashita T, Yamawaki S, Ishii S (2002). Complications associated with bone cementing for the treatment of giant cell tumors of bone. J Orthop Sci..

[22] Sanzana ES, Navarro M, Ginebra MP, Planell JA, Ojeda AC, Montecinos HA (2014). Role of porosity and pore architecture in the in vivo bone regeneration capacity of biodegradable glass scaffolds. J Biomed Mater Res A..

[23] Torres-Sanchez C, Al Mushref FRA, Norrito M, Yendall K, Liu Y, Conway PP (2017). The effect of pore size and porosity on mechanical properties and biological response of porous titanium scaffolds. Mater Sci Eng C Mater Biol Appl..

[24] Mahjoub M, Berenbaum F, Houard X (2012). Why subchondral bone in osteoarthritis? The importance of the cartilage bone interface in osteoarthritis. Osteoporos Int..

[25] Teng W, Lin P, Li Y, Yan X, Li H, Li B, Wang Z, Wu Y, Wang S, Zhou X, Wang Z, Ye Z (2019). Bone combined cement grafting in giant cell tumor around the knee reduces mechanical failure. Int Orthop..

